# A distinct epigenetic signature at targets of a leukemia protein

**DOI:** 10.1186/1471-2164-8-38

**Published:** 2007-02-01

**Authors:** Stefano Rossetti, André T Hoogeveen, Ping Liang, Cornel Stanciu, Peter van der Spek, Nicoletta Sacchi

**Affiliations:** 1 Department of Cancer Genetics, Roswell Park Cancer Institute, Elm and Carlton Streets, Buffalo, NY 14263, USA; 2 Department of Clinical Genetics, Erasmus MC, Dr Molewaterplein 50, 3015GE Rotterdam, The Netherlands; 3 Department of Bioinformatics, Erasmus MC, Dr Molewaterplein 50, 3015GE Rotterdam, The Netherlands

## Abstract

**Background:**

Human myelogenous leukemia characterized by either the non random t(8; 21)(q22; q22) or t(16; 21)(q24; q22) chromosome translocations differ for both their biological and clinical features. Some of these features could be consequent to differential epigenetic transcriptional deregulation at AML1 targets imposed by AML1-MTG8 and AML1-MTG16, the fusion proteins deriving from the two translocations. Preliminary findings showing that these fusion proteins lead to transcriptional downregulation of AML1 targets, marked by repressive chromatin changes, would support this hypothesis. Here we show that combining conventional global gene expression arrays with the power of bioinformatic genomic survey of AML1-consensus sequences is an effective strategy to identify AML1 targets whose transcription is epigenetically downregulated by the leukemia-associated AML1-MTG16 protein.

**Results:**

We interrogated mouse gene expression microarrays with probes generated either from 32D cells infected with a retroviral vector carrying AML1-MTG16 and unable of granulocyte differentiation and proliferation in response to the granulocyte colony stimulating factor (G-CSF), or from 32D cells infected with the cognate empty vector. From the analysis of differential gene expression alone (using as criteria a p value < 0.01 and an absolute fold change > 3), we were unable to conclude which of the 37 genes downregulated by AML1-MTG16 were, or not, direct AML1 targets. However, when we applied a bioinformatic approach to search for AML1-consensus sequences in the 10 Kb around the gene transcription start sites, we closed on 17 potential direct AML1 targets. By focusing on the most significantly downregulated genes, we found that both the AML1-consensus and the transcription start site chromatin regions were significantly marked by aberrant repressive histone tail changes. Further, the promoter of one of these genes, containing a CpG island, was aberrantly methylated.

**Conclusion:**

This study shows that a leukemia-associated fusion protein can impose a distinct epigenetic repressive signature at specific sites in the genome. These findings strengthen the conclusion that leukemia-specific oncoproteins can induce non-random epigenetic changes.

## Background

Nuclear hormone receptors and transcription factors can regulate the transcription of their target genes by inducing chromatin changes. Paradigmatic are the retinoic acid receptor alpha (RARα) and the transcription factor core binding factor (CBF), which regulate in this way the transcription of target genes involved in hematopoietic processes [1,2]. Differently from RARα, which epigenetically activates its targets by recruiting coactivator protein complexes with histone acetyl transferase (HAT) activity only when bound to retinoic acid, CBF can directly recruit HAT-containing complexes to activate its targets [3-6]. One of the two CBF subunits, CBFα or AML1, can bind target genes endowed with the AML1-consensus sequence TG(T/C)GGT via its N-terminal DNA-binding domain [7]. *AML1*, encoding a master hematopoietic transcription factor, is frequently affected by different chromosome translocations in leukemic cells [8]. Moreover, *AML1 *haploinsufficiency was found to be associated with familial platelet disorder, a condition predisposing to acute myeloid leukemia [9].

Two leukemia-associated chromosome translocations, the t(8;21)(q22;q22) and the t(16;21)(q24;q22), result in the fusion between the N-terminal region of AML1 and the C-terminal regions of two almost identical chromatin corepressors, MTG8 and MTG16, leading to the formation of AML1-MTG8 and AML1-MTG16, respectively [10-13]. Upon fusion with either MTG8 or MTG16, AML1 is converted from a transcriptional activator into a transcriptional repressor of AML1-targets. Specific MTG domains in the wild type, as well as in the MTG fusion proteins, can interact, directly or via other corepressors such as NCoR and Sin3A, with histone deacetylases (HDACs), thus creating a repressive chromatin state at AML1 target sites (reviewed in [14,15]). Repression at these sites is further enhanced by the formation of oligomers between the fusion proteins and wild-type MTG proteins [16-18].

Myeloid cell differentiation systems, such as the 32D mouse myeloid cell line, ectopically expressing either AML1-MTG8 or AML1-MTG16, were used as models to simulate some of the effects of these fusion proteins in myelogenesis and leukemogenesis. Both fusion proteins, when exogenously expressed in the 32D background, were shown to affect granulocytic differentiation and produce distinct effects on cell proliferation [19-21]. In a preliminary study, we found that AML1-MTG16, when exogenously expressed in 32D cells, can induce aberrant myeloid phenotypes in association with repressive modifications at the chromatin of the Colony stimulating factor 1 receptor (*Csf1r*), an AML1-target gene encoding the macrophage colony stimulating factor receptor [19]. Based on this finding, we hypothesize that the comparative epigenetic analysis of the changes induced by different AML1-MTG fusion proteins in an identical cell context (e.g. the 32D context) might provide a lead to elucidating the differences observed in leukemic cells carrying either one of the two proteins [8]. The objective of this study was to demonstrate whether AML1-MTG16 induces epigenetic changes at AML1-target genes in the 32D myeloid cell genome. Only by coupling global gene expression array analysis with a bioinformatic genomic survey for the AML1-consensus sequence, we were able to close onto AML1-targets downregulated by AML1-MTG16. AML1-MTG16-induced transcriptional downregulation was marked by the acquisition of a distinct repressive chromatin signature.

## Results

### Global gene expression array analysis of AML1-MTG16-expressing cells

**Figure 1:**
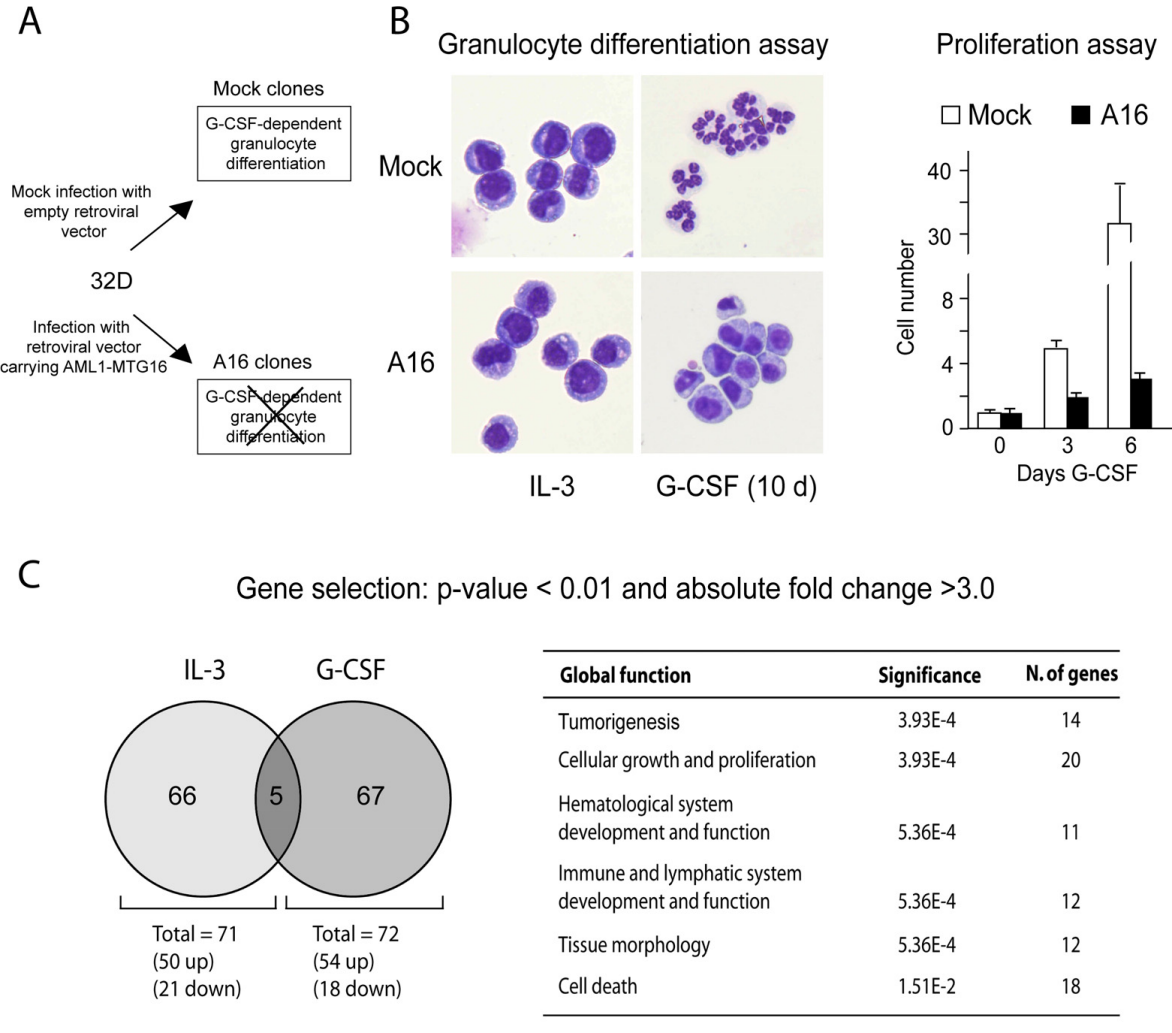
**Global gene expression analysis of AML1-MTG16-expressing cells**. **A**. The 32D cell model, comprising clones expressing the AML1-MTG16 protein (A16 clones) and control clones ("mock" clones), which do not express the fusion protein. **B**. A16 clones, differently from mock clones, do not undergo granulocytic differentiation and display an impaired proliferation in the presence of G-CSF. **C**. Most of the genes whose expression is significantly affected in A16 cells were found previously implicated in biological processes.

To study the molecular and biological consequences of AML1-MTG16 expression in a myeloid differentiation cell model, we previously developed, by infecting 32D mouse myeloblasts with retroviral particles carrying either the pLNCX2 vector containing the AML1-MTG16 cDNA or the cognate empty vector, stable independent clones expressing AML1-MTG16 (hereafter called A16 clones) and stable independent control clones (hereafter called "mock" clones), respectively (Figure 1A). Upon treatment with granulocyte colony stimulating factor (G-CSF), A16 clones do not undergo granulocytic differentiation and proliferate significantly less than mock clones (Figure 1B). Global gene expression analysis (setting the p-value at < 0.05 and the absolute fold change at > 1.5) of a prototypic A16 clone and a prototypic mock clone grown either with interleukin 3 (IL-3) or G-CSF for 16 h, was combined with bioinformatic analysis of the proteins encoded by all the differentially expressed genes with the Ingenuity software (see Methods). This analysis clearly revealed a network comprising proteins critical for platelet function in A16 cells (see Additional file 1). The identification of this protein network strongly supports the biological data, indicating the occurrence of functional AML1 haploinsufficiency in A16 cells [9].

Further analysis of the gene expression data (setting the p-value at < 0.01 and the absolute fold change at > 3) enabled us to identify 138 differentially expressed genes, of which 66 differentially expressed genes in cells grown with IL-3, 67 differentially expressed genes in cells grown with G-CSF, and 5 differentially expressed genes in both cells grown with IL-3 and G-CSF (Figure 1C, left, and Table 1 and Table 2). According to the Ingenuity software, the differentially expressed genes in A16 cells were mostly implicated in tumorigenesis, cell proliferation, and hematopoiesis (Figure 1C, right). Since from this analysis alone we were unable to conclude whether, or not, these genes were AML1-MTG16 direct targets, we devised a bioinformatic approach aimed at identifying the AML1-consensus sequence in the 10 Kb region around the transcription start site of these genes.

### Identification of genes containing the AML1-consensus sequence by bioinformatic analysis

**Figure 2:**
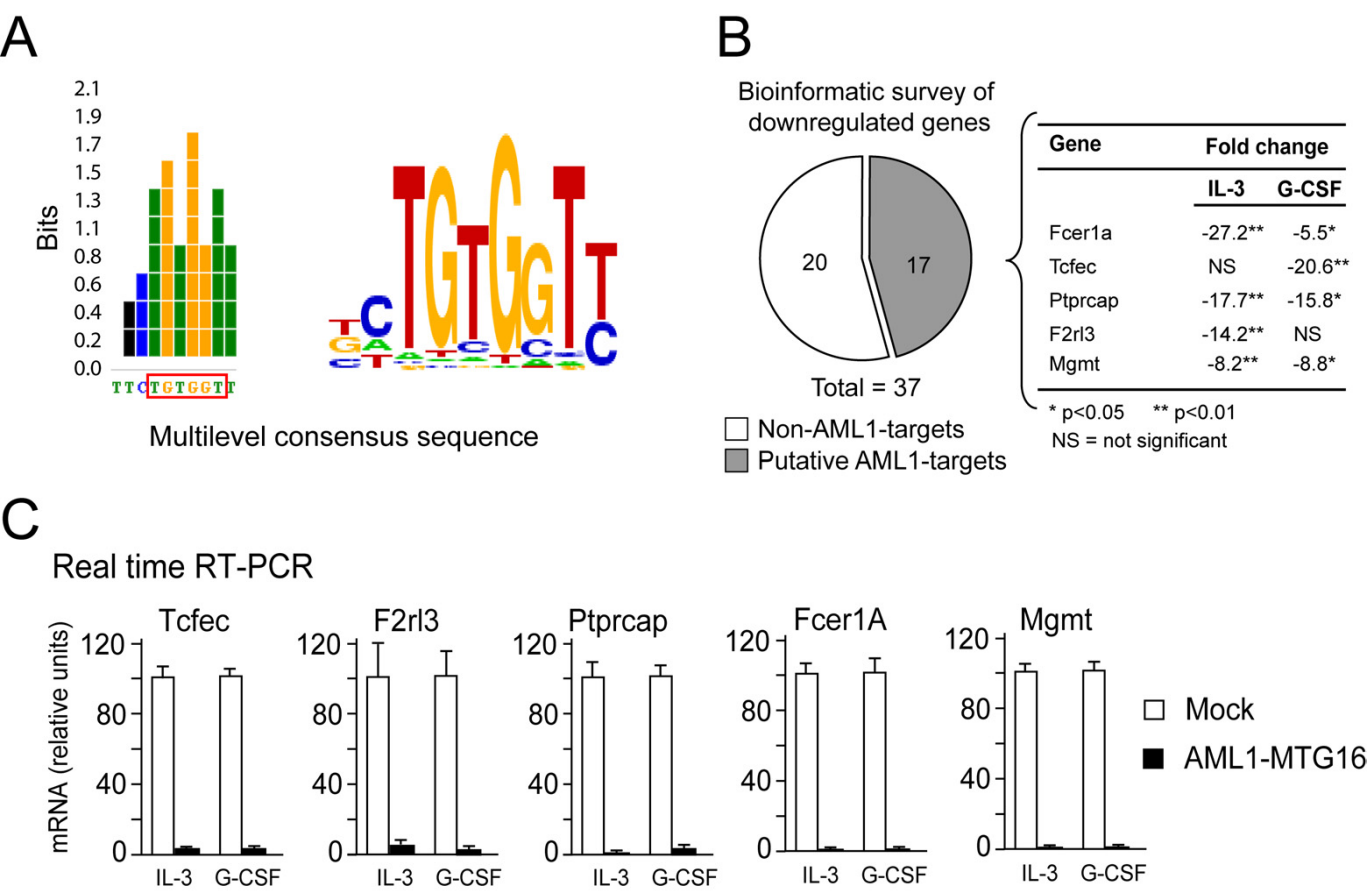
**AML1-MTG16-induced downregulation of putative AML1-targets**. **A**. The AML1-consensus motif, containing the AML1-consensus sequence (framed), found by bioinformatic analysis of the genes significantly downregulated in A16 cells. The height of the columns associated with each nucleotide is proportional to the conservation level. The "logo" representation of the motif instead indicates in which proportion the single bases are present at each position. **B**. Seventeen out of the 37 downregulated genes are putative AML1-targets. The fold-changes of five of the most significantly downregulated genes are reported at right. **C**. Real time RT-PCR confirmed the significant (p < 0.01) downregulation of the five genes.

Since the AML1-MTG proteins have a transcriptionally repressive function (reviewed in [14]), we focused our bioinformatic analysis on the 37 genes downregulated by AML1-MTG16 (see genes in bold in Table 1 and Table 2). Specifically, we searched the 10 Kb around the transcription start site of each gene for either the AML1-binding consensus sequence TG(T/C)GGT or, this sequence in reverse orientation, ACC(G/A)CA. With the MEME software (see Methods) we identified a conserved motif, hereafter called AML1-consensus motif (Figure 2A), encompassing the AML1-consensus sequence in seventeen out of the 37 genes (Figure 2B and Table 3). We focused on five of these genes, *Fcer1a, Tcfec, Ptprcap, F2rl3*, and *Mgmt *(Figure 2B, right), because they were among the most significantly downregulated genes. *Fcer1a, Tcfec, Ptprcap, F2rl3*, and *Mgmt *encode for known proteins. Specifically, Fcer1a is the Fc fragment of IgE and is involved in the immune response [22]; Tcfec is a transcription factor that induces, among other genes, the G-CSF receptor gene [23,24]; Ptprcap is a transmembrane protein associated with CD45, a key regulator of lymphocytes activation [25]; F2rl3 is a member of G protein-coupled protease-activated receptors (PARs) of the coagulation factor II (thrombin) and plays an important role in platelet activation [26]; Mgmt is a DNA repair enzyme that is frequently lost in cancer due to epigenetic silencing [27]. Downregulation of these genes was confirmed by real time RT-PCR (Figure 2C).

### *Fcer1a, Tcfec, Ptprcap, F2rl3*, and *Mgmt *are direct AML1-MTG16 targets

**Figure 3:**
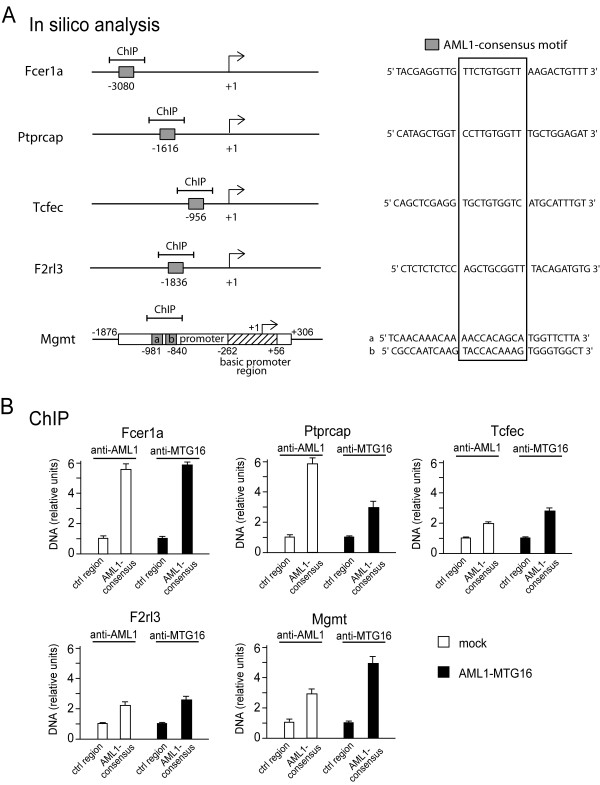
**AML1-target gene validation**. **A**. Relative position of the AML1-consensus motifs (left) and their sequence (right) in the five putative AML1-target genes that were analyzed by ChIP. **B**. Quantitative ChIP analysis with antibodies either against AML1 or MTG16 showing a significant (p < 0.05) enrichment of chromatin containing AML1-consensus motifs vs. chromatin containing a control region in AML1-MTG16-negative and AML1-MTG16-positive cells, respectively.

Quantitative chromatin immunoprecipitation (ChIP) with an anti-AML1 specific antibody, but not with an anti-MTG16 antibody (data not shown), showed significant (p < 0.05) enrichment of the region encompassing the AML1-consensus motif (see bars in figure 3A, left) relative to an arbitrary control region without the AML1-consensus motif in the mock clone chromatin for all five genes, indicating endogenous AML1 binding at these regions (Figure 3B). ChIP with an anti-MTG16 antibody showed instead a significant enrichment of exogenous AML1-MTG16 in the same chromatin regions in the A16 clones (Figure 3B). The human homologues of these genes also contain an AML1-consensus sequence(s) in the 10Kb region surrounding the transcription start site, pointing to these five genes as novel, *bona fide *direct AML1-targets genes.

### Repressive chromatin changes at AML1-MTG16-downregulated targets

**Figure 4:**
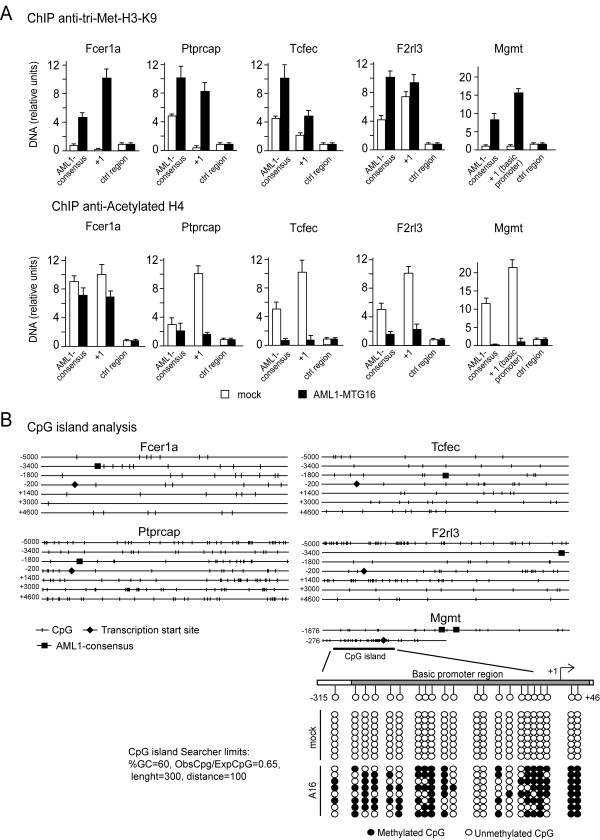
**Repressive epigenetic changes at the AML1-targets**. **A**. ChIP with antibodies against either acetylated histone H4 or tri-methylated histone H3 Lysine 9 (tri-Met-H3-K9) followed by quantitative PCR with primers amplifying a region encompassing either the transcription start site (+1) or the AML1-consensus detected a different level of repressive histone changes in all five genes in A16 cells. **B**. *In silico *analysis identified a CpG island only in the *Mgmt *promoter. This CpG island is hypermethylated in A16 cells (bottom, right).

We previously demonstrated that AML1-MTG16 interacts with both HDAC1 and HDAC3 [28]. Further, we found that AML1-MTG16 can induce downregulation marked by repressive histone hypoacetylation at the *Csf1r *chromatin [19]. Here we show that, in A16 cells, the chromatin associated with both the region containing the AML1-consensus motif and the region encompassing the transcription start site of *Fcer1a, Tcfec, Ptprcap, F2rl3*, and *Mgmt *(Figure 3A) displays a significant (p < 0.05) decrease of acetylated histone H4 (Ac-H4), and a significant (p < 0.05) increase of H3K9 tri-methylation (Tri-Met-H3-K9) (Figure 4A), supporting the acquisition of a repressive chromatin state [29-31].

Repressive histone modifications are often associated with aberrant hypermethylation at CpG islands present in the 5' regulatory regions of many genes [32,33] and references within). By using the CpG island searcher [34], a software for the identifying CpG islands, we could identify a CpG island only in the *Mgmt *promoter region [35] (Figure 4B). Bisulfite sequencing analysis of this region detected hypermethylation in AML1-MTG16-positive cells (Figure 4B).

The overall epigenetic analysis indicates that downregulation of AML1-targets by AML1-MTG16 can be achieved, even in the absence of DNA methylation, when there is a critical quantitative level of repressive histone changes.

## Discussion

In this study we show the effectiveness of integrating global gene expression array analysis with a bioinformatic approach aimed at detecting AML1-consensus sequences for identifying novel putative direct AML1-targets downregulated by AML1-MTG16 in 32D cells. Downregulation of these genes is marked by a distinct repressive chromatin profile.

When we surveyed the 37 most significantly downregulated genes for the presence of the AML1-consensus motif(s) in the 10 Kb region encompassing the transcription start site, we closed on seventeen putative direct AML1-MTG16 targets. For five of these genes, *Fcer1a, Tcfec, Ptprcap, F2rl3 *and *Mgmt*, which were among the most significantly downregulated, we were able to demonstrate, using ChIP analysis, the binding of both AML1 and AML1-MTG16 to the gene regions containing the AML1-motifs. Thus, our two-tier approach, combining gene expression array analysis with bioinformatic survey for transcription factor-consensus sequences, seems to be a powerful strategy for identifying transcription factor targets, which would otherwise be missed when using conventional gene expression array analysis alone.

The chromatin of the five downregulated genes, *Fcer1a, Tcfec, Ptprcap, F2rl3*, and *Mgmt*, was marked not only by significant levels of histone H4 hypoacetylation, but also by significant levels of repressive histone H3-K9 trimethylation, suggesting that AML1-MTG16 might induce the recruitment of both histone deacetylases [28] and histone methyltransferases. Apparently, a critical quantity of repressive histone modifications, even in the absence of CpG methylation, might *per se *be sufficient to "lock in" a transcriptionally downregulated state. In the case of *Mgmt*, which has a CpG island, it is instead possible that the accumulation of histone repressive changes preceded CpG hypermethylation [[36], and references within].

It is noteworthy that all the genes for which we demonstrated AML1-MTG16-induced epigenetic downregulation encode for functions relevant to either hematopoiesis and/or leukemogenesis. We would like to underline that downregulation of two of the genes that we identified might be relevant to AML1-MTG16-induced leukemogenesis. One of these genes is *Tcfec*, whose human counterpart encodes a transcription factor that induces the granulocyte colony stimulating factor receptor *G-CSFR *[23,24]. Remarkably, *Tcfec *downregulation in A16 cells is paralleled by a significant downregulation of *G-csfr *(data not shown), indicating that AML1-MTG16 might have triggered a coordinated cascade of transcriptional downregulation, as we observed in other differentiation model systems [37,38]. The second gene is *Mgmt*, encoding the DNA repair enzyme O6-Methylguanine-DNA-methyltransferase, which is frequently silenced and hypermethylated in leukemia [39]. *MGMT *epigenetic silencing is thought to lead to random mutations in cancer [40]. A recent study has shown that expression of different acute myeloid leukemia fusion proteins, including AML1-MTG8, leads to downregulation of several DNA repair genes [41]. Thus, the induction of a "mutator phenotype" might be a common consequence of leukemia fusion protein expression.

A few global gene expression studies on cells expressing exogenous AML1-MTG8 have been recently described [42-44]. Given the use of different cell systems, it is difficult to compare the differentially expressed genes in AML1-MTG16-positive 32D cells with the differentially expressed genes reported for AML1-MTG8. Nevertheless, we could identify a few gene families (e.g. S100 Calcium-binding proteins) that are similarly affected by both AML1-MTG8 and AML1-MTG16 even in different cell contexts. Extending our study to the comparison of the epigenetic signatures imposed by either exogenous AML1-MTG16 or exogenous AML1-MTG8 in the very same cell context (e.g. 32D cells) might enable us to narrow down additional critical epigenetic signatures consequent to t(8;21) and t(16;21) translocations.

## Conclusion

In this study, we show that AML1-MTG16, the leukemia fusion protein associated with the non-random chromosome translocation t(16;21)(q24;q22), can impose transcriptional downregulation marked by a distinct epigenetic signature at specific AML1-target sites in the genome. Thus, our findings further support the hypothesis that non-random genetic abnormalities can lead to non-random epigenetic changes in leukemia cells [19,45].

## Methods

### Cell cultures

Stable clones obtained from mouse myeloid 32D cells infected either with pLNCX2-AML1-MTG16 (A16 clones) or the empty vector pLNCX2 (mock clones) were previously described [19]. Two prototypic A16 clones and two prototypic mock clones were used in this study. Cells were maintained in the presence of 10 ng/ml of murine IL-3 (BD Biosciences, San Jose, CA, USA) in RPMI 1640 medium supplemented with 10% fetal calf serum, 1% antibiotics (penicillin/streptomycin), adjusting the cell density to 2 × 10^5 ^cells/ml daily. To induce granulocyte differentiation, cells were washed in RPMI medium, and IL-3 was replaced with 10 ng/ml human G-CSF (Amgen, Thousand Oaks, CA, USA). Differentiation was microscopically evaluated on cytospin preparations stained with May-Grünwald-Giemsa.

### RNA extraction and microarray hybridization

Total RNA was extracted with RNeasy mini kit (Qiagen, Hilden, Germany) and treated with DNase (Qiagen). Double stranded cDNA was generated from 5 μg RNA using Superscript ds cDNA synthesis kit (Invitrogen, Carlsbad, CA, USA) and T7-oligo(dT) primers. The cDNA was purified with GeneChip Sample Cleanup Module (Affymetrix, Santa Clara, CA, USA) and used to synthesize biotin-labeled cRNA with Enzo RNA transcript Labeling Kit (Enzo Life Science, Farmingdale, NY, USA). Purified cRNA was quantified by spectrophotometric methods and the concentration was adjusted in order to exclude the carryover of unlabeled RNA. 11 μg of cRNA were then fragmented in fragmentation buffer (Affymetrix) at 95°C for 35 minutes and hybridized for 16 h at 45°C onto MOE430A microarrays (Affymetrix). After washing and staining, the chips were scanned in a Hewett-Packard/Affymetrix scanner at 570 nm. For all the samples the 5'/3' ratios of *Gapdh *were 0.7 – 0.9. In comparative experiments the scaling factor, noise and presence calls were similar. Gene expression data represent the average of two independent experiments.

### Microarray data analysis

The arrays were normalized by geometric mean intensity for each probe set and scaled using log2 transformation for further analysis. Comparison between the A16 and mock clones grown with either IL-3 or G-CSF was done using Spotfire Decision Site. This comparison generated a p-value from a t-test to statistically extract significant changes in mRNA expression levels between the groups. p-values < 0.05 were considered significant. The null hypothesis is that the samples between the groups are derived from the same population i.e. there is no significant differential expression. The t-test looks at the variance within the groups as well as between them. To be considered significantly differentially expressed the variance had to be greater between than within the groups to a level of p < 0.05. Ratios were generated by dividing the average of the unlogged control data by the average of the unlogged AML1-MTG16 data. Ratios were then portrayed as positive or negative fold change between A16 and mock. To confirm statistical significance of these ratios the differentially expressed genes had to satisfy an arbitrary cut-off ratio as well as having a p-value < 0.05 (see Results section). Analysis of the protein networks was performed by using Ingenuity Pathways Analysis (Ingenuity Systems, Redwood City, CA), software able to identify molecular networks based on known functional or physical interactions among the proteins encoded by the differentially expressed genes.

### Search of AML1-consensus sequence in differentially expressed genes

The well-annotated genes differentially expressed in the A16 clone *versus *the mock clone either in the presence of IL-3 or G-CSF (p < 0.01 and absolute fold change >3) were searched for the AML1-consensus sequence "5'-TG(T/C)GGT-3"' in the 10 kb region surrounding the transcription initiation sites (from -5000 bp to +5000 bp) using an in-house built PERL script. A 400 bp sequence flanking the potential AML1-binding sites (200 bp on each side) was extracted and analyzed with MEME, which is a software package to discover motifs in groups of related DNA sequences [46], and with multiple sequence alignment to test whether additional conserved motifs in the surrounding regions could be identified and to assess the sequence conservation extending the potential AML1-binding sites.

### Real-time RT-PCR

Total RNA was obtained using Trizol (Invitrogen), treated with DNase I (Ambion, Austin, TX, USA), retrotranscribed with SuperScript™ First-Strand Synthesis System (Invitrogen) and amplified by Real-time RT-PCR on an iCycler (Bio-Rad, Hercules, CA, USA) by using iQ SYBR Green Supermix (Bio-Rad) and primers specific for *γ actin*, *F2rl3*, *Fcer1a*, *Ptprcap*, *Tcfec*, and *Mgmt *(Table 4). Transcript levels of the genes of interest were quantitated by the Delta-delta Ct method, using the house keeping gene *γ-actin *for normalization. The amplification efficiency, evaluated from the sample slopes, was similar for all the samples analyzed in the same experiment. Two independent experiments were performed in triplicate using two mock clones and two A16 clones. Significance was determined by using the Student t-test.

### Quantitative chromatin immunoprecipitation (ChIP)

ChIP was performed using reagents purchased from Upstate (Charlottesville, VA, USA) following the manufacturer's protocol. AML1 and AML1-MTG16 binding was assessed by ChIP with antibodies against either the AML1 C-terminus (Santa Cruz Biotechnology, Santa Cruz, CA, USA), or the MTG16 C-terminus [28], respectively. Histone hallmarks of repressive chromatin were assessed by ChIP with antibodies against acetyl-histone H4 (Upstate) and trimethyl-K9 at histone H3 (Upstate). Control ChIPs were performed without the respective antibodies. The immunoprecipitated DNA was amplified by real-time PCR with primers specific for regions encompassing the AML1-consensus, the transcription start site, or a control region (Table 4). The DNA relative enrichment was calculated by using the Delta-delta Ct method. The PCR signals obtained for each gene region were normalized to the PCR signal obtained from the input DNA (total chromatin fraction) and compared to a control region approximately 15 kb downstream of *F2rl3 *transcription start site. Two independent experiments were performed in triplicate, and significance was calculated by using the Student t-test.

### Bisulfite sequencing

Genomic DNA was extracted with DNAzol (Invitrogen) according to the manufacturer's instructions. DNA was modified by sodium bisulfite treatment as previously described [47]. *Mgmt *CpG island was amplified by nested PCR by using the primers indicated in Table 4. The PCR fragments were subcloned into pGEM-T (Promega, San Luis Obispo, CA, USA) and 20 clones for each PCR fragment were sequenced.

## Authors' contributions

SR developed the 32D clones, designed and carried out the molecular genetics studies, participated in the microarray analysis, and prepared a draft of the manuscript. ATH contributed to the microarray analysis and critically reviewed the manuscript. PL performed the bioinformatic genome search of AML1-motifs. CS provided technical help for the real time RT-PCR and ChIP analyses. PV performed the microarray data analysis. NS conceived the hypothesis and critically reviewed the entire manuscript. All authors read and approved the final manuscript.

## Supplementary Material

Additional File 1 Evidence of functional AML1 haploinsufficiency in AML1-MTG16-expressing cells. This figure shows the Ingenuity Pathways Analysis of the global gene expression changes identified in AML1-MTG16-expressing cells. Click here for file

## Figures and Tables

**Table 1:** Selection of genes differentially expressed in AML1-MTG16-positive cells versus AML1-MTG16-negative cells grown in the presence of IL-3.

**Affymetrix ID**	**NCBI acc. number**	**Gene Symbol**	**Gene Title**	**GO/Ingenuity annotations**	**Fold change**
1450042_at	BB322201	*Arx*	aristaless related homeobox gene (Drosophila)	regulation of transcription	16.4
1460300_a_at	NM_008523	*Ltk*	leukocyte tyrosine kinase	kinase signaling pathway	15.9
1423869_s_at	AF349659	*Txnrd3*	thioredoxin reductase 3	electron transport	12.9
1418796_at	NM_009131	*Scgf*	stem cell growth factor	cell adhesion/cell proliferation	9.6
1427329_a_at	AI326478	*Igh-6*	immunoglobulin heavy chain 6 (heavy chain of IgM)	immune response	8.6
1418588_at	NM_009513	*Vmp*	vesicular membrain protein p24	---	7.4
1450652_at	NM_007802	*Ctsk*	cathepsin K	proteolysis	7.2
1428439_at	BG066220	*Nyren18-pending*	NY-REN-18 antigen	---	6.4
1419416_a_at	NM_011244	*Rarg*	retinoic acid receptor, gamma	regulation of transcription	6.3
1426800_at	BM214169	*D330025I23Rik (Cbfb)*	RIKEN cDNA D330025I23 gene (core-binding factor beta subunit)	regulation of transcription	6.2
1419136_at	NM_134066	*Akr1c18*	aldo-keto reductase family 1, member C18	electron transport	6.1
1425432_at	AF260307	*Oprm*	opioid receptor, mu	G-protein signaling pathway	6.0
1418346_at	NM_013754	*Insl6*	insulin-like 6	physiological processes	6.0
1449426_a_at	NM_011922	*Anxa10*	annexin A10	---	5.9
1423029_at	NM_008236	*Hes2*	hairy and enhancer of split 2 (Drosophila)	regulation of transcription	5.8
1454007_a_at	AK020384	*Zfp142*	zinc finger protein 142	electron transport	5.8
1423313_at	BG070255	*Pde7a*	phosphodiesterase 7A	signal transduction	5.8
1451915_at	L20509	*Cct3*	chaperonin subunit 3 (gamma)	protein folding	5.7
1452487_x_at	BB133664	*Pirb*	paired-Ig-like receptor B	---	5.7
1422030_at	AF326316	*Atp6v0a4*	ATPase, H+ transporting, lysosomal V0 subunit A isoform 4	ATP hydrolysis/proton transport	5.6
1427753_at	Z95479	*Igh-4*	immunoglobulin heavy chain 4 (serum IgG1)	immune response	5.5
1437235_x_at	BB218844	*Lpp*	LIM domain containing preferred translocation partner in lipoma	cytoskeleton organization/transcriptional regulation	5.4
1426938_at	BB627486	*Nova1*	neuro-oncological ventral antigen 1	mRNA splicing	5.0
1460416_s_at	M55219	*Csprs*	component of Sp100-rs	G-protein signaling pathway	4.9
1427884_at	AW550625	*Col3a1*	procollagen, type III, alpha 1	cell adhesion	4.9
1450453_a_at	NM_012065	*Pde6g*	phosphodiesterase 6G, cGMP-specific, rod, gamma	vision	4.8
1455957_x_at	AV034167	*Ceacam11*	CEA-related cell adhesion molecule 11	---	4.7
1450215_at	NM_009038	*Rcvrn*	recoverin	vision	4.7
1452489_at	BC016258	*Vps11*	vacuolar protein sorting 11 (yeast)	protein transport	4.4
1421705_at	NM_018732	*Scn3a*	sodium channel, voltage-gated, type III, alpha polypeptide	ion transport	4.4
1421375_a_at	NM_011313	*S100a6*	S100 calcium binding protein A6 (calcyclin)	cell proliferation	4.4
1433658_x_at	AV300794	*Pcbp4*	poly(rC) binding protein 4	apoptosis	4.2
1418136_at	NM_009365	*Tgfb1i1*	transforming growth factor beta 1 induced transcript 1	regulation of transcription	4.2
1450629_at	AV114522	*Eplin-pending*	epithelial protein lost in neoplasm	---	3.9
1455421_x_at	AW490145	*Clcn1*	chloride channel 1	ion transport	3.7
1418451_at	BB522409	*Gng2*	guanine nucleotide binding protein (G protein), gamma 2 subunit	G-protein signaling pathway	3.7
1450709_at	NM_007851	*Defcr5*	defensin related cryptdin 5	defense response	3.5
1423561_at	AI838010	*Nell2*	nel-like 2 homolog (chicken)	cell adhesion	3.4
1452279_at	BB800282	*Pfc*	properdin factor, complement	complement activation	3.4
1424531_a_at	BC010807	*Tcea3*	transcription elongation factor A (SII), 3	regulation of transcription	3.4
1419325_at	NM_019515	*Nmu*	neuromedin	neuropeptide signaling pathway	3.4
1422945_a_at	AI844677	*Kif5c*	kinesin family member 5C	protein transport	3.3
1460280_at	NM_010815	*Mona*	monocytic adaptor	intracellular signaling cascade	3.3
1448529_at	NM_009378	*Thbd*	thrombomodulin	blood coagulation	3.2
1449830_at	NM_013766	*Prlpi*	prolactin-like protein I	---	3.2
1423596_at	BB528391	*Nek6*	NIMA (never in mitosis gene a)-related expressed kinase 6	kinase signaling pathway/cell proliferation	3.2
1450435_at	NM_008478	*Slc7a2*	solute carrier family 7 (cationic amino acid transporter, y+ system), member 2	amino acid transport	3.2
1420373_at	BI249549	*Foxj2*	forkhead box J2	regulation of transcription	3.1
1436769_at	AV101011	*Psma1*	proteasome (prosome, macropain) subunit, alpha type 1	ubiquitin-dependent protein catabolism	3.1
1421778_at	NM_011911	*V1rb2*	vomeronasal 1, receptor B2	chemosensory perception/G-protein signaling pathway	3.0
**1448416_at**	**NM_008597**	***Mglap***	**matrix gamma-carboxyglutamate (gla) protein**	**---**	**-3.0**
**1419012_at**	**NM_011766**	***Zfpm2***	**zinc finger protein, multitype 2**	**regulation of transcription **	**-3.0**
**1449833_at**	**NM_011472**	***Sprr2f***	**small proline-rich protein 2F**	**---**	**-3.1**
**1424814_a_at**	**BC025541**	***9030625M01Rik (Bclg)***	**RIKEN cDNA 9030625M01 gene (apoptosis regulator Bclg)**	**apoptosis**	**-3.1**
**1417338_at**	**U03487**	***Epb4.2***	**erythrocyte protein band 4.2**	**structural function**	**-3.3**
**1448152_at**	**NM_010514**	***Igf2***	**insulin-like growth factor 2**	**cell proliferation **	**-3.6**
**1429947_a_at**	**AK008179**	***Zbp1***	**Z-DNA binding protein 1**	**---**	**-3.7**
**1420394_s_at**	**U05264**	***Gp49b***	**glycoprotein 49 B**	**immune response?**	**-3.7**
**1424898_at**	**BC021154**	***Slc10a1***	**solute carrier family 10 (sodium/bile acid cotransporter family), member 1**	**ion transport**	**-3.8**
**1416822_at**	**BC013711**	***Es2el***	**expressed sequence 2 embryonic lethal**	**---**	**-4.0**
**1420779_at**	**NM_010213**	***Fhl3***	**four and a half LIM domains 3**	**cytoskeleton organization **	**-4.3**
**1419124_at**	**NM_133829**	***AW212394***	**expressed sequence AW212394**	**---**	**-4.4**
**1425597_a_at**	**AW060288**	***Qk***	**quaking**	**apoptosis**	**-4.6**
**1422416_s_at**	**NM_016983**	***Vpreb2***	**Pre-B lymphocyte gene 2**	**hematopoiesis**	**-4.7**
**1425863_a_at**	**AF295638**	***Ptpro***	**protein tyrosine phosphatase, receptor type, O**	**phosphatase signaling pathway**	**-4.8**
**1418177_at**	**AF233778**	***Gabrg2***	**gamma-aminobutyric acid (GABA-A) receptor, subunit gamma 2**	**synaptic transmission **	**-4.8**
**1421309_at**	**NM_008598**	***Mgmt***	**O-6-methylguanine-DNA methyltransferase**	**DNA repair**	**-8.2**
**1421288_at**	**NM_007975**	***F2rl3***	**coagulation factor II (thrombin) receptor-like 3**	**blood coagulation/G-protein signaling pathway**	**-14.2**
**1449347_a_at**	**NM_021365**	***Xlr4***	**X-linked lymphocyte-regulated 4**	**chromatin remodeling?**	**-16.9**
**1448511_at**	**NM_016933**	***Ptprcap***	**protein tyrosine phosphatase, receptor type, C polypeptide-associated protein**	**phosphatase signaling pathway**	**-17.7**
**1421775_at**	**NM_010184**	**Fcer1a**	**Fc receptor, IgE, high affinity I, alpha polypeptide**	**signal transduction**	**-27.2**

Limits: p-value < 0.01; absolute fold change > 3.

In bold are the AML1-MTG16-downregulated genes searched for AML1-consensus motifs.

**Table 2:** Selection of genes differentially expressed in AML1-MTG16-positive cells versus AML1-MTG16-negative cells grown in the presence of G-CSF for 16 h.

**Affymetrix ID**	**NCBI acc. number**	**Gene Symbol**	**Gene Title**	**GO/Iingenuity annotations**	**Fold change**
1437100_x_at	BB206220	*Pim3*	proviral integration site 3	kinase signaling pathway	24.5
1460300_a_at	NM_008523	*Ltk*	leukocyte tyrosine kinase	kinase signaling pathway	19.9
1416257_at	NM_009794	*Capn2*	calpain 2	proteolysis/cell migration	17.7
1417314_at	NM_008198	*H2-Bf*	histocompatibility 2, complement component factor B	cell proliferation/complement activation	14.7
1425380_at	AF331457	*Rasgrp4*	RAS guanyl releasing protein 4	intracellular signaling cascade	10.4
1450322_s_at	NM_011409	*Slfn3*	schlafen 3	cell proliferation	10.2
1421793_at	NM_010198	*Fgf11*	fibroblast growth factor 11	signal transduction/cell proliferation	9.5
1420348_at	NM_008499	*Lhx5*	LIM homeobox protein 5	regulation of transcription	8.8
1419605_at	NM_010796	*Mgl1*	macrophage galactose N-acetyl-galactosamine specific lectin 1	cell adhesion	8.6
1420360_at	NM_010051	*Dkk1*	dickkopf homolog 1 (Xenopus laevis)	signal transduction/apoptosis	6.7
1425647_at	BG069740	*Rnf33*	ring finger protein 33	---	6.4
1434851_s_at	AU015319	*Crb3*	crumbs homolog 3 (Drosophila)	intercellular junction assembly	6.1
1427102_at	AF099975	*Slfn4*	schlafen 4	cell proliferation	5.9
1437218_at	BM234360	*Fn1*	fibronectin 1	cell adhesion	5.5
1417777_at	BC014865	*Ltb4dh*	leukotriene B4 12-hydroxydehydrogenase	metabolism	5.5
1419406_a_at	NM_016707	*Bcl11a*	B-cell CLL/lymphoma 11A (zinc finger protein)	T/B-cell differentiation/corepressor	5.5
1418358_at	NM_008574	*Mcsp*	mitochondrial capsule selenoprotein	sperm motility	5.4
1450499_at	NM_009124	*Sca1*	spinocerebellar ataxia 1 homolog (human)	---	5.2
1418257_at	BB732135	*Slc12a7*	solute carrier family 12, member 7	ion transport	5.1
1424744_at	BC021950	*Sds*	serine dehydratase	amino acid metabolism	5.1
1456305_x_at	BB702568	*Obox1*	oocyte specific homeobox 1	regulation of transcription	5.0
1449707_at	C80272	*Nr5a2*	nuclear receptor subfamily 5, group A, member 2	regulation of transcription	4.9
1421504_at	NM_009239	*Sp4*	trans-acting transcription factor 4	regulation of transcription	4.8
1427079_at	U51204	*Mapre3*	microtubule-associated protein, RP/EB family, member 3	cytoskeleton organization	4.8
1429626_at	AV024301	*Sftpa*	surfactant associated protein A	cell adhesion	4.8
1452793_at	AI509011	*Cldn10*	claudin 10	cell adhesion	4.7
1419507_at	NM_013713	*Krtap15*	keratin associated protein 15	---	4.7
1421375_a_at	NM_011313	*S100a6*	S100 calcium binding protein A6 (calcyclin)	cell proliferation	4.5
1419517_at	NM_028408	*2900075G08Rik*	RIKEN cDNA 2900075G08 gene	intracellular signaling cascade	4.4
1454736_at	BM119297	*4921515A04Rik*	RIKEN cDNA 4921515A04 gene	regulation of transcription	4.3
1436244_a_at	AU067681	*Tle2*	transducin-like enhancer of split 2, homolog of Drosophila E(spl)	regulation of transcription/signal transduction	4.2
1420594_at	NM_007525	*Bard1*	BRCA1 associated RING domain 1	DNA repair/regulation of transcription/apoptosis	4.2
1426093_at	AF220141	*Trim34*	tripartite motif protein 34	---	4.2
1424748_at	BC021504	*Galnt11*	UDP-N-acetyl-alpha-D-galactosamine:polypeptide N-acetylgalactosaminyltransferase 11	metabolism	4.1
1416855_at	BB550400	*Gas1*	growth arrest specific 1	cell cycle arrest///programmed cell death	4.0
1422310_at	NM_009223	*Snn*	stannin	---	4.0
1452463_x_at	BG966217	*Igk-V8*	immunoglobulin kappa chain variable 8 (V8)	immune response	4.0
1450415_at	NM_008805	*Pde6a*	phosphodiesterase 6A, cGMP-specific, rod, alpha	signal transduction	3.9
1418792_at	AF326561	*Sh3gl2*	SH3-domain GRB2-like 2	---	3.9
1451759_at	BC013893	*Masp2*	mannan-binding lectin serine protease 2	cell adhesion/complement activation	3.9
1418921_at	AY059393	*Necl1-pending*	nectin-lke 1	cell adhesion	3.9
1416188_at	BC004651	*Gm2a*	GM2 ganglioside activator protein	sphingolipid metabolism	3.8
1448392_at	NM_009242	*Sparc*	secreted acidic cysteine rich glycoprotein	cell proliferation	3.8
1419485_at	BB759833	*Foxc1*	forkhead box C1	regulation of transcription	3.7
1419602_at	NM_010451	*Hoxa2*	homeo box A2	regulation of transcription	3.7
1427358_a_at	BC026671	*Dapk1*	death associated protein kinase 1	apoptosis	3.6
1450827_at	NM_024245	*Kif23*	kinesin family member 23	mitosis	3.6
1421280_at	Z36357	*Gabra1*	gamma-aminobutyric acid (GABA-A) receptor, subunit alpha 1	synaptic transmission	3.5
1452279_at	BB800282	*Pfc*	properdin factor, complement	complement activation	3.5
1415854_at	BB815530	*Kitl*	kit ligand	cell proliferation/cell adhesion	3.4
1417513_at	AI255184	*Evi5*	ecotropic viral integration site 5	---	3.3
1431379_a_at	AK005153	*Slc13a1*	solute carrier family 13 (sodium/sulphate symporters), member 1	ion transport	3.2
1418476_at	NM_018827	*Crlf1*	cytokine receptor-like factor 1	---	3.2
1416009_at	NM_019793	*Tm4sf8-pending*	transmembrane 4 superfamily member 8	signal transduction/cell proliferation	3.1
**1451633_a_at**	**BC025929**	***Gng1***	**guanine nucleotide binding protein (G protein), gamma 1 subunit**	**G-protein signaling pathway**	**-3.0**
**1425978_at**	**AF384055**	***Srfcp-pending***	**SRF co-factor protein (cardiac and smooth muscle)**	**regulation of transcription/positive regulation of cell proliferation**	**-3.0**
**1425153_at**	**BC008538**	***Myh2***	**myosin, heavy polypeptide 2, skeletal muscle, adult**	**cytoskeleton organization**	**-3.1**
**1448755_at**	**AF011450**	***Col15a1***	**procollagen, type XV**	**cell adhesion**	**-3.2**
**1433888_at**	**AV343478**	***Atp2b2***	**ATPase, Ca++ transporting, plasma membrane 2**	**metabolism**	**-3.5**
**1426868_x_at**	**AK003174**	***Lmna***	**lamin A**	**cell morphology**	**-3.5**
**1423292_a_at**	**BG072867**	***Prx***	**periaxin**	**intracellular signaling cascade**	**-3.6**
**1449891_a_at**	**NM_028523**	***Esdn-pending***	**endothelial and smooth muscle cell-derived neuropilin-like molecule**	**---**	**-3.6**
**1425708_at**	**AF285585**	***Rnf17***	**ring finger protein 17**	**---**	**-4.2**
**1449836_x_at**	**NM_007546**	***Biklk***	**Bcl2-interacting killer-like**	**apoptosis**	**-4.6**
**1448710_at**	**D87747**	***Cxcr4***	**chemokine (C-X-C motif) receptor 4**	**defense response/hematopoiesis**	**-4.8**
**1419227_at**	**NM_009839**	***Cct6b***	**chaperonin subunit 6b (zeta)**	**protein folding**	**-5.0**
**1455853_x_at**	**BB768303**	***2700085A14Rik (Sas)***	**RIKEN cDNA 2700085A14 gene (Sarcoma amplified sequence)**	**cell proliferation/signal transduction**	**-5.3**
**1416822_at**	**BC013711**	***Es2el***	**expressed sequence 2 embryonic lethal**	**---**	**-5.4**
**1422473_at**	**BM246564**	***Pde4b***	**phosphodiesterase 4B, cAMP specific**	**signal transduction**	**-7.8**
**1418499_a_at**	**NM_020574**	***Kcne3***	**potassium voltage-gated channel, Isk-related subfamily, gene 3**	**ion transport**	**-8.3**
**1419537_at**	**NM_031198**	***Tcfec***	**transcription factor EC**	**regulation of transcription**	**-20.6**
**1449347_a_at**	**NM_021365**	***Xlr4***	**X-linked lymphocyte-regulated 4**	**chromatin remodeling?**	**-34.4**

Limits: p-value < 0.01; absolute fold change > 3.

In bold are the AML1-MTG16-downregulated genes searched for AML1-consensus motifs.

**Table 3:** Selection of putative AML1-target genes downregulated in AML1-MTG16-positive cells versus AML1-MTG16-negative cells.

**Affymetrix ID**	**NCBI acc. number**	**Gene Symbol**	**Gene Title**	**GO/Ingenuity annotations**	**Fold change IL-3**	**Fold change G-CSF**
1417338_at	U03487	Epb4.2	erythrocyte protein band 4.2	structural function	-3.3	---
1433888_at	AV343478	Atp2b2	ATPase, Ca++ transporting, plasma membrane 2	metabolism	---	-3.5
1426868_x_at	AK003174	Lmna	lamin A	cell morphology	---	-3.5
1423292_a_at	BG072867	Prx	periaxin	intracellular signaling cascade	---	-3.6
1449891_a_at	NM_028523	Esdn-pending	endothelial and smooth muscle cell-derived neuropilin-like molecule	---	---	-3.6
1425708_at	AF285585	Rnf17	ring finger protein 17	---	---	-4.2
1419124_at	NM_133829	AW212394	expressed sequence AW212394	---	-4.4	---
1425597_a_at	AW060288	Qk	quaking	apoptosis	-4.6	---
1419227_at	NM_009839	Cct6b	chaperonin subunit 6b (zeta)	protein folding	---	-5.0
1455853_x_at	BB768303	2700085A14Rik (Sas)	RIKEN cDNA 2700085A14 gene (Sarcoma amplified sequence)	cell proliferation/signal transduction	---	-5.3
1422473_at	BM246564	Pde4b	phosphodiesterase 4B, cAMP specific	signal transduction	---	-7.8
1421309_at	NM_008598	*Mgmt*	O-6-methylguanine-DNA methyltransferase	DNA repair	-8.2	---
1421288_at	NM_007975	F2rl3	coagulation factor II (thrombin) receptor-like 3	blood coagulation/G-protein signaling pathway	-14.2	---
1449347_a_at	NM_021365	Xlr4	X-linked lymphocyte-regulated 4	chromatin remodelling?	-16.9	-34.4
1448511_at	NM_016933	Ptprcap (1)	protein tyrosine phosphatase, receptor type, C polypeptide-associated protein	phosphatase signaling pathway	-17.7	---
1419537_at	NM_031198	Tcfec	transcription factor EC	regulation of transcription	---	-20.6
1421775_at	NM_010184	Fcer1a	Fc receptor, IgE, high affinity I, alpha polypeptide	signal transduction	-27.2	---

Motif conservation significance: p < 10E-5.

(1) The *Ptprcap *AML1-consensus motif is located in an intron of a 5' adjacent gene (*Coro1b*).

**Table 4:** Primers used for real time RT-PCR, quantitative ChIP, and bisulfite sequencing.

**Primer name**	**Orientation**	**Sequence**
***Real time PCR primers***		

γ-Actin	sense	5'-GCCGGCTTACACTGCGCTTCTT-3'
	antisense	5'-TTCTGGCCCATGCCCACCAT-3'
F2rl3	sense	5'-GCTTCTGATCCTGGCAGCATG-3'
	antisense	5'-GTGTCACTGTCGTTGGCACAG-3'
Fcer1a	sense	5'-CCCTTTCCTGCTATGGGAACA-3'
	antisense	5'-GCAGCCAATCTTGCGTTACATT-3'
Ptprcap	sense	5'-GGATGAAGAGGATGCAGAAGAT-3'
	antisense	5'-CTGACTCCTATAGTGCAGTGAC-3'
Tcfec	sense	5'-AGTCTAATGATCCTGATATGCGC-3'
	antisense	5'-TCCTGAATCCGGAGCCTAAGC-3'
Mgmt	sense	5'-GAACTTGGCAGAATGGCTGAG-3'
	antisense	5'-GGTGATGGAGAGCAGGCAA-3'

***ChIP primers***		

Ptprcap- AML1-consensus	sense	5'-GTCCTGCAGCTGGTGTTTACAG-3'
	antisense	5'-CTGGTCTCTGAGTGGCTGCA-3'
Ptprcap-transcription start	sense	5'-GAGGTCTGACAAGTTAGCTGTA-3'
	antisense	5'-ACCCTGTAACTCACTTCTCACT-3'
Tcfec- AML1-consensus	sense	5'AGAGCTTGACTAGAATGGATTT-3'
	antisense	5'-GGTGCAACCCATTCATGGCTT-3'
Tcfec-transcription start	sense	5'-AGTCACACCACTGGAGTAGTTTT-3'
	antisense	5'-CCCTCGTCTCATAACCTAAGCA-3'
Fcer1a- AML1-consensus	sense	5'-GGCCACTGACTTCAGTGTGAA-3'
	antisense	5'-TGCATTCCAGTTCTCTGCAAGA-3'
Fcer1a-transcription start	sense	5'-AGGTGTCAGCTGAAGGTACAATA-3'
	antisense	5'-CCCACCATGACACTCTCTAAAT-3'
F2rl3-AML1-consensus	sense	5'-AGGGTGTCTCTCTGAATCTGGA-3'
	antisense	5'-GGCAAGTCTGTTATCTCAGCAT-3'
F2rl3-transcription start	sense	5'-TTGGAGGAAGGCTGGATTGTTAT-3'
	antisense	5'-CCCATTGGGATCTGCTTGCTCA-3'
Mgmt-AML1-consensus	sense	5'-GAGCTGCACACTGGGAAGATG-3'
	antisense	5'-GTGTACCAGATGCTGTGCAGG-3'
Mgmt-basic promoter	sense	5'-CAGTTTCAGGTCTGGAAGAAGAG-3'
	antisense	5'-AGCTGTGGGCTTGTAGTCCGAG-3'
Control region	sense	5'-ATGCAACACACAACAAAGCAAA-3'
	antisense	5'-GGCCAAATGAGGTTGTGTCCT-3'

***Bisulfite sequencing primers***		

Mgmt-CpG-1^st ^PCR	sense	5'-TAGTGATTGGATTTTTAGTGGGT-3'
	antisense	5'-CTATCTCCCTAAACTTCAACTC-3'
Mgmt-CpG-2^nd ^PCR	sense	5'-GTGAGAAGGTGTAGTTTAGTTT-3'
	antisense	5'-CTCACCAACTTACAAACTACAA-3'
