# Supplementary material for: A distinct epigenetic signature at targets of a leukemia protein
Source: BMC Genomics. 2007 Feb 1;8:38. doi: 10.1186/1471-2164-8-38 (PMC1796549; doi:10.1186/1471-2164-8-38)
Supplement: Additional File 1 — Evidence of functional AML1 haploinsufficiency in AML1-MTG16-expressing cells. This figure shows the Ingenuity Pathways Analysis of the global gene expression changes identified in AML1-MTG16-expressing cells. [file 1471-2164-8-38-S1.pdf]

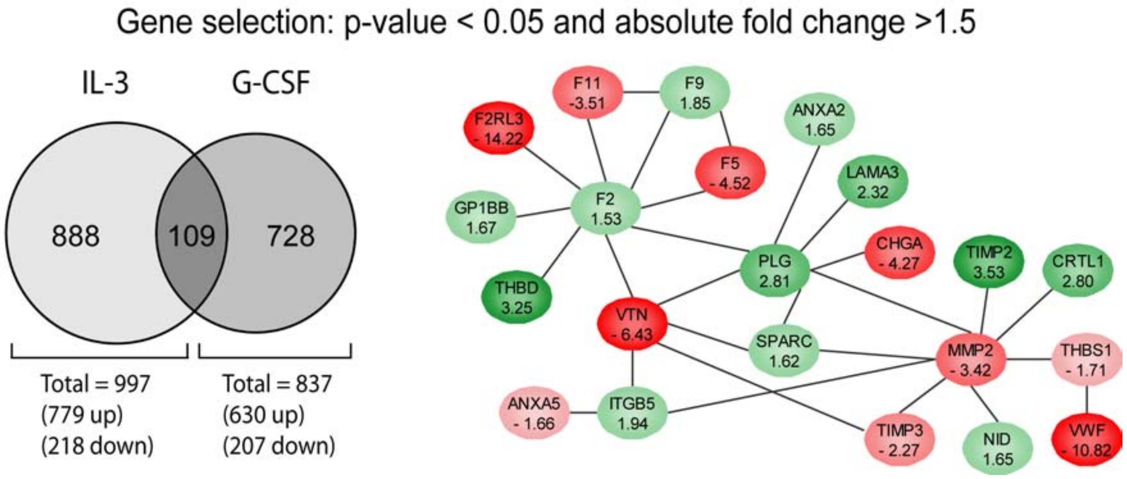

Additional File 1 - Evidence of functional AML1 haploinsufficiency in AML1-MTG16-expressing cells. Global gene expression analysis (setting $p < 0.05$ and absolute fold change > 1.5) identified 997 and 837 genes differentially expressed in A16 cells in the presence of IL-3 and G-CSF, respectively (left). Identification of a protein network related to platelet formation and blood coagulation in A16 cells provides evidence of the occurrence of functional AML1 haploinsufficiency (right).
